# Genetic and biochemical characterizations of Zika virus NS2A protein

**DOI:** 10.1080/22221751.2019.1598291

**Published:** 2019-04-08

**Authors:** Xianwen Zhang, Xuping Xie, Jing Zou, Hongjie Xia, Chao Shan, Xinwen Chen, Pei-Yong Shi

**Affiliations:** a State Key Laboratory of Virology, Wuhan Institute of Virology, Chinese Academy of Sciences, Wuhan, People’s Republic of China; b University of Chinese Academy of Sciences, Beijing, People’s Republic of China; c Department of Biochemistry & Molecular Biology, University of Texas Medical Branch, Galveston, TX, USA; d Guangzhou Institute of Biomedicine and Health, Chinese Academy of Sciences, Guangzhou, People’s Republic of China; e Institute for Human Infections & Immunity, University of Texas Medical Branch, Galveston, TX, USA; f Sealy Institute for Vaccine Sciences, University of Texas Medical Branch, Galveston, TX, USA; g Sealy Center for Structural Biology & Molecular Biophysics, University of Texas Medical Branch, Galveston, TX, USA; h Department of Pharmacology & Toxicology, University of Texas Medical Branch, Galveston, TX, USA

**Keywords:** Zika virus, flavivirus replication, virion assembly, flavivirus NS2A, membrane topology

## Abstract

Zika virus (ZIKV) can cause devastating congenital Zika syndromes in pregnant women and Guillain-Barre syndrome in adults. Understanding the molecular mechanism of ZIKV replication is essential for antiviral and vaccine development. Here we report the structural and functional characterization of ZIKV NS2A protein. Biochemical structural probing suggests that ZIKV NS2A has a single segment that traverses the ER membrane and six segments that peripherally associate with the ER membrane. Functional analysis has defined distinct NS2A residues essential for viral RNA synthesis or virion assembly. Only the virion assembly-defective mutants, but not the RNA synthesis-defective mutants, could be rescued through *trans* complementation with a wide-type NS2A protein. These results suggest that the NS2A molecules in virion assembly complex could be recruited in *trans*, whereas the NS2A molecules in viral replication complex must be recruited in *cis*. Together with previous results, we propose a flavivirus assembly model where NS2A plays a central role in modulating viral structural and nonstructural proteins as well as genomic RNA during virion assembly.

## Introduction

Zika virus (ZIKV) is a recently emerged mosquito-borne flavivirus that is primarily transmitted by *Aedes spp.* mosquitoes. It can also be transmitted through sexual, maternal-to-fetal, and blood transfusion routes [1–5]. ZIKV was first identified in 1947 from a sentinel rhesus monkey in Uganda [6]. Since 2007, it emerged in the Pacific region, South America, Central America, and the Caribbean, posing a global public health threat [7–11]. ZIKV infection causes mild illnesses, including fever, pruritis, arthritis, conjunctivitis, and asthenia [12]. Importantly, it can cause devastating congenital Zika syndromes (CZS; including microcephaly, congenital malformation, and fetal demise) in pregnant women and Guillain-Barre syndrome (GBS) in adults [13–16]. The explosive epidemics and its association with CZS had led the World Health Organization (WTO) to declare ZIKV as a Public Health Emergency of International Concern from February to November in 2016 [17]. Since then, a number of vaccine candidates have been developed, some of which have already advanced to clinical trials [18]. In addition, small molecule inhibitors and therapeutic antibodies have also been identified for antiviral development [19–22].

ZIKV belongs to the *Flavivirus* genus within the *Flaviviridae* family. Besides ZIKV, many other flaviviruses are significant human pathogens, including dengue (DENV), yellow fever (YFV), West Nile (WNV), Japanese encephalitis (JEV), Saint Louis encephalitis (SLEV), and tick-borne encephalitis virus (TBEV) [23]. Flavivirus genome is a positive-sense, single-stranded RNA of ∼11,000 nucleotides. It contains a 5’ untranslated region (UTR), a long open-reading-frame (ORF), and a 3’ UTR. The single ORF encodes a polyprotein that is processed into three structure proteins (capsid [C], pre-membrane [prM] and envelope [E]) and seven nonstructural proteins (NS1, NS2A, NS2B, NS3, NS4A, NS4B, and NS5). The structural proteins, together with genomic RNA, are components of virions. The nonstructural proteins participate in viral RNA replication, virion assembly, and evasion of host immune response [24–26].

Flavivirus NS2A is an ER-resident, transmembrane protein with a calculated molecular weight of ∼22 kDa. The N-terminus and C-terminus of NS2A are formed by cleavages with an unknown host protease and viral protease NS3 (in complex with NS2B), respectively [27,28]. Flavivirus NS2A has multiple functions: (i) Viral replication. Kunjin virus (KUNV) NS2A co-localizes with viral double-stranded RNA and interacts with the 3’UTR of genomic RNA, NS3, and NS5 protein [29]. Mutagenesis studies confirmed the role of NS2A in viral RNA synthesis [30,31]. (ii) Virion assembly/secretion. Mutagenesis analyses of YFV, KUNV, and DENV have identified distinct NS2A amino acids that are essential for virion assembly/secretion [30–34]. The virion assembly defects can be restored by second site mutations in NS2B or NS3 in YFV and DENV [32,35,36]. (iii) Evasion of innate immunity. ZIKV NS2A inhibits type-I IFN induction at the step of TBK1 [26]. DENV NS2A antagonizes type-I IFN signalling [25]. JEV NS2A blocks dsRNA-activated protein kinase PKR [37]. In WNV and KUNV, the NS2A-mediated inhibition of type-I IFN induction can be abrogated by specific NS2A mutations [38,39]. (iv) Disease pathogenesis. WNV NS2A modulates virus-induced cytopathic effect (CPE) and pathogenesis in mice [40,41]. ZIKV NS2A impairs mammalian cortical neurogenesis through depleting adherens junction proteins [42].

The structure and function of ZIKV NS2A have not been characterized. Although NS2A proteins from different flaviviruses perform similar functions (described above), each flavivirus may exert its NS2A functions in a virus-specific manner [35]. The goals of this study are (i) to define the membrane topology of ZIKV NS2A and (ii) to characterize its roles in viral RNA synthesis and virion assembly.

## Materials and methods

### Cell lines

Vero cells, baby hamster kidney (BHK-21) cells and human embryo kidney 293T cells were cultured at 37°C with 5% CO_2_ in high-glucose Dulbecco’s modified Eagle medium (DMEM; Life Technologies) supplemented with 2 mM L-glutamine, 100 U/ml penicillin, 100 g/ml streptomycin and 10% fetal bovine serum (FBS; HyClone Laboratories).

### Bioinformatics

NS2A protein sequences from ZIKV, DENV1, DENV2, DENV3, DENV4, WNV, JEV, YFV, SLEV were aligned using CLC Main Workbench software (CLC Bio). Prediction of ZIKV NS2A was carried out using various web servers, which including TOPCONS, Philius, Polyphobius, SCAMPI, and TMHMM2. The amphipathic helices of ZIKV NS2A were predicted by HeliQuest.

### Antibody

The following antibodies were used in this study: a mouse monoclonal antibody 4G2 cross-reactive with flavivirus E protein (American Type Culture Collection); Rabbit anti-HA monoclonal antibody (Cell Signaling Technology); Alexa Fluor®488 conjugated mouse anti-HA monoclonal antibody (Cell Signaling Technology); Rabbit anti-Calnexin polyclonal antibody(Abcam); Mouse anti-GFP monoclonal antibody (Sigma-Aldrich); Horseradish peroxidase(HRP) conjugated goat anti-mouse and anti-rabbit IgG polyclonal antibodies (Sigma-Aldrich); Alexa Fluor®488 conjugated goat anti-mouse IgG polyclonal antibody (Thermo Fisher Scientific); Alexa Fluor®568 conjugated goat anti-rabbit IgG polyclonal antibody (Thermo Fisher Scientific).

### Plasmid construction

The infectious cDNA clone (pFLZIKV) bearing the full-length genome of ZIKV (strain FSS13025) or Renilla luciferase (Rluc) reporter ZIKV replicon (pZIKV Rep WT) were used as the backbone for introducing NS2A mutations [43,44]. XhoI and KpnI restriction sites were initially generated into pFLZIKV and pZIKV Rep WT replicon at positions 3018 and 5010 in ZIKV genome without changing amino acid sequences. The NS2A alanine-scanning mutagenesis was performed by using a QuikChange II XL Site-Directed Mutagenesis kit (Agilent Technologies) as described previously [30].

A mammalian expression vector pXJ with a cytomegalovirus (CMV) promoter was used for constructing plasmids expressing NS2A and its derivatives. To construct the pXJ-E_24_-NS1-NS2A-HA plasmid, a gene cassette encoding the last 24 amino acids of E protein (E_24_), NS1 and NS2A with a C-terminal hemagglutinin (HA) tag was firstly amplified from the pFLZIKV and cloned into the pXJ vector. Next, NS2A mutations were individually introduced into the pXJ-E_24_-NS1-NS2A-HA by overlap PCR, resulting in plasmids expressing NS2A mutants.

To construct the pXJ-SPG-C_16_-NS2A-HA that was used for selecting cell line constitutively expressing NS2A protein, a DNA fragment containing the Gaussia luciferase signal peptide (SPG), the last 16 amino acids of NS1 protein (C_16_) and NS2A tagged with a C-terminal HA tag sequence were constructed by overlap PCR and cloned into the pXJ vector via NotI/XhoI restriction sites.

Plasmid pXJ-eGFP was constructed by inserting the enhanced green fluorescence protein (eGFP) sequence into the pXJ vector via BamHI/XhoI restriction sites. DNA fragments that encode different NS2A segments were individually inserted into above pXJ-eGFP vector, resulting various pXJ-smNS2A-eGFP plasmids (smNS2A represents NS2A segment). A DNA fragment that express NS2A or truncated NS2A and an eGFP with a C-terminal N-glycosylation acceptor site (Glyc) were amplified and cloned into the pXJ-SPG-C_16_-NS2A-HA vector, resulting in the plasmid pXJ-SPG-C_16_-NS2A-eGFP-Glyc and various pXJ-SPG-C_16_-ctNS2A-eGFP-Glyc plasmids (ctNS2A represents C-terminal truncated NS2A).

### RNA in vitro transcription and electroporation

All full-length cDNA clone plasmids and subgenomic replicons of ZIKV were linearized with restriction enzyme ClaI, followed by phenol-chloroform extraction and ethanol precipitation. RNA transcripts were prepared by using the T7 mMessage mMachine kit (Ambion). For RNA transfection, 10 μg *in vitro* transcribed RNA was electroporated into 8 × 10^6^ cells (BHK-21 or Vero) by using Gene Pulser Xcell™ Electroporation Systems (Bio-rad) as described previously [30,43].

### Immunofluorescence assay

For indirect immunofluorescence assay (IFA), cells were seeded in an 8-well Nunc Lab-Tek II Chamber Slide (Thermo Fisher Scientific). At various time points, cells were washed three times with phosphate-buffered saline (PBS), fixed with 4% paraformaldehyde in PBS at room temperature for 30 min and permeabilized with 0.1% Triton X-100 in PBS at room temperature for 10 min. The permeabilized cells were then washed three times with PBS and incubated in a blocking buffer containing 1% FBS in PBS at room temperature for 1 h. E protein was stained with a 1:1000 dilution of mouse monoclonal antibody 4G2 in blocking buffer. After incubation for 1 h, the cells were washed three times with a washing buffer (1% FBS and 0.5% Tween 20 in PBS). After 1 h of incubation with a secondary antibody (Alexa Fluor®488 conjugated goat anti-mouse IgG), the cells were washed three times with washing buffer and counterstained with DAPI (4’,6-diamidino-2-phenylindole). Images were acquired using an Olympus fluorescence microscope. Images were processed in ImageJ software (National Institutes of Health, MD).

### Plaque assay

2 × 10^5^ Vero cells per well were grown in 24-well plates one day prior to viral infection. 10-fold serial dilutions of virus samples were prepared in DMEM containing 2% FBS and 1% penicillin/streptomycin and inoculated (100 µl) into each well of 24-well plates. After 1 h of incubation at 37°C with 5% CO_2_, the inoculum was replaced with 500 µl overlay medium (DMEM medium containing 0.8% methyl-cellulose, 2% FBS and 1% penicillin/streptomycin). Plaques formed after incubation at 37°C with 5% CO_2_ for 4–5 days. Cells were fixed with 4% paraformaldehyde for 30 min, followed by staining with 1% crystal violet. The numbers of plaque per millilitre (PFU/ml) of ZIKV were calculated.

### Replicon transient-transfection assay

Briefly, 8 × 10^6^ Vero cells were electroporated with 10 μg wild type or mutant Rluc-ZIKV replicon RNAs. After electroporation, 3 × 10^5^ cells per well were seeded in 12-well plates. At given time points, the cells were washed twice with PBS and lysed in 200 μl of luciferase lysis buffer (Promega). The luciferase activity assay was performed according to the manufacturer’s protocol.

### RNA extraction and cDNA sequencing

The culture fluids from infected cells were centrifuged at 1000 g for 10 min and then filtered through a 0.22 μm pore size polyethersulfone membrane (Millipore) to remove cellular debris. Viral RNAs in the supernatants were extracted using a QIAamp viral-RNA Minikit (Qiagen) by following the manufacturer’s protocol. Reverse transcription PCR (RT-PCR) was performed by using the SuperScript® III One-Step RT-PCR System (Thermo Fisher Scientific). The amplified cDNA fragments were purified and sent for Sanger sequencing at the GENEWIZ facilities.

### Selection of NS2A-HA BHK-21 cell line

4 × 10^5^ BHK-21 cells per well were seeded into a 6-well plate. The plasmid pXJ-SPG-C_16_-NS2A-HA that contains a neomycin resistance gene was transfected into BHK-21 cells (4 µg per well) using X-tremeGENE 9 DNA transfection reagent (Roche). At 24 h post-transfection, geneticin (G418) was added to the culture medium with a concentration of 1 mg/ml. The medium was changed every three days. After two weeks, several neomycin-resistant individual colonies were selected for propagation. The expression of NS2A-HA protein in stable cell lines was analyzed by immunofluorescence assay and Western blot.

### In vitro proteinase K protection assay

Proteinase K protection assay was performed as previously described with minor modifications [45,46]. BHK-21 cells were transfected in 6-well plates with various pXJ-SPG-C_16_-ctNS2A-eGFP-Glyc plasmids. After incubated at 30°C for 24 h, cells were trypsinized, washed twice with PBS and centrifuged at 450 × g for 5 min. The cell pellets were resuspended in a hypotonic lysis buffer (10 mM Tris-HCl, pH 7.5, and 2 mM MgCl_2_) and kept on ice for 10 min. Subsequently, the samples were homogenized with 40 strokes and centrifuged at 1000 × g for 5 min. The postnuclear supernatants (PNS) were divided into three aliquots and centrifuged at 15,000 × rpm for 40 min. The pellets from three aliquots were then resuspended in PBS, 60 μg/ml proteinase K in PBS, or 60 μg/ml proteinase K in PBS in the presence of 0.5% Triton X-100, respectively. The samples were incubated on ice for 90 min. 20 mM phenylmethylsulfonyl fluoride (PMSF) was finally added to terminate the reaction. After further incubation on ice for 15 min, the samples were mixed with 4× Laemmli sample buffer, heated at 95°C for 15 min and analyzed by Western blotting.

### In vitro deglycosylation assay

The samples were prepared by following a previously described protocol [46,47]. Briefly, the transfected BHK-21 cells in 6-well plates were incubated at 30°C for 24 h. Cells were trypsinized and suspended in DMEM medium containing 10%FBS. The cells were pelleted by centrifuging at 450 × g for 5 min. After two PBS washes, the cell pellets were resuspended in a solubilization buffer (20 mM Tris-HCl, pH 7.5, 50 mM NaCl, 10 mM Mg(CH_3_COO)_2_, 1% Triton X-100, and EDTA-free protease inhibitors [Roche]) and incubated at 4°C for 2 h with gentle agitation. After centrifugation at 15,000 rpm 4°C for 20 min to remove cell debris, the supernatants were split into two aliquots. 50,000 units/ml N-glycosidase F (PNGase F) or PBS (no-treatment control) were added to those two aliquots. After incubation at 37°C for 2 h, the samples were mixed with 4× Laemmli sample buffer, heated at 70°C for 15 min and analyzed by Western blot.

### SDS-PAGE and Western blot

Protein samples were separated by 12% SDS-polyacrylamide gel electrophoresis and transferred onto a polyvinylidene difluoride (PVDF) membrane using Trans-Blot Turbo transfer system (Bio-Rad), after which the membrane was blocked in TBST (10 mM Tris-HCl, pH 8.0, 150 mM NaCl, and 0.1% Tween 20) supplemented with 5% nonfat milk for 1 h at room temperature. The blots were probed with primary antibodies (1:1,000 dilution) overnight at 4°C, washed three times with TBST and incubated with horseradish peroxidase (HRP) conjugated goat anti-mouse and anti-rabbit IgG polyclonal antibodies (1:10,000 dilution) for 1 h. After three thorough TBST buffer washes, the blots were incubated with SuperSignal™ West Femto Maximum Sensitivity Substrate (Thermo Fisher Scientific). Chemiluminescence signals were detected in ChemiDoc System (Bio-Rad).

### Quantification and statistical analysis

IFA images processing and cell counting were performed in software ImageJ (NIH). Densitometry analysis was performed using Image lab version 6.0 (Bio-Rad Laboratories, Hercules, CA). All numerical data are presented as the mean ± SEM (standard error of mean). The size of each study or number of replicates, along with the statistical tests performed can be found in corresponding Figure Legends.

## Results

### The rationale for ZIKV NS2A mutagenesis

**Figure 1.**
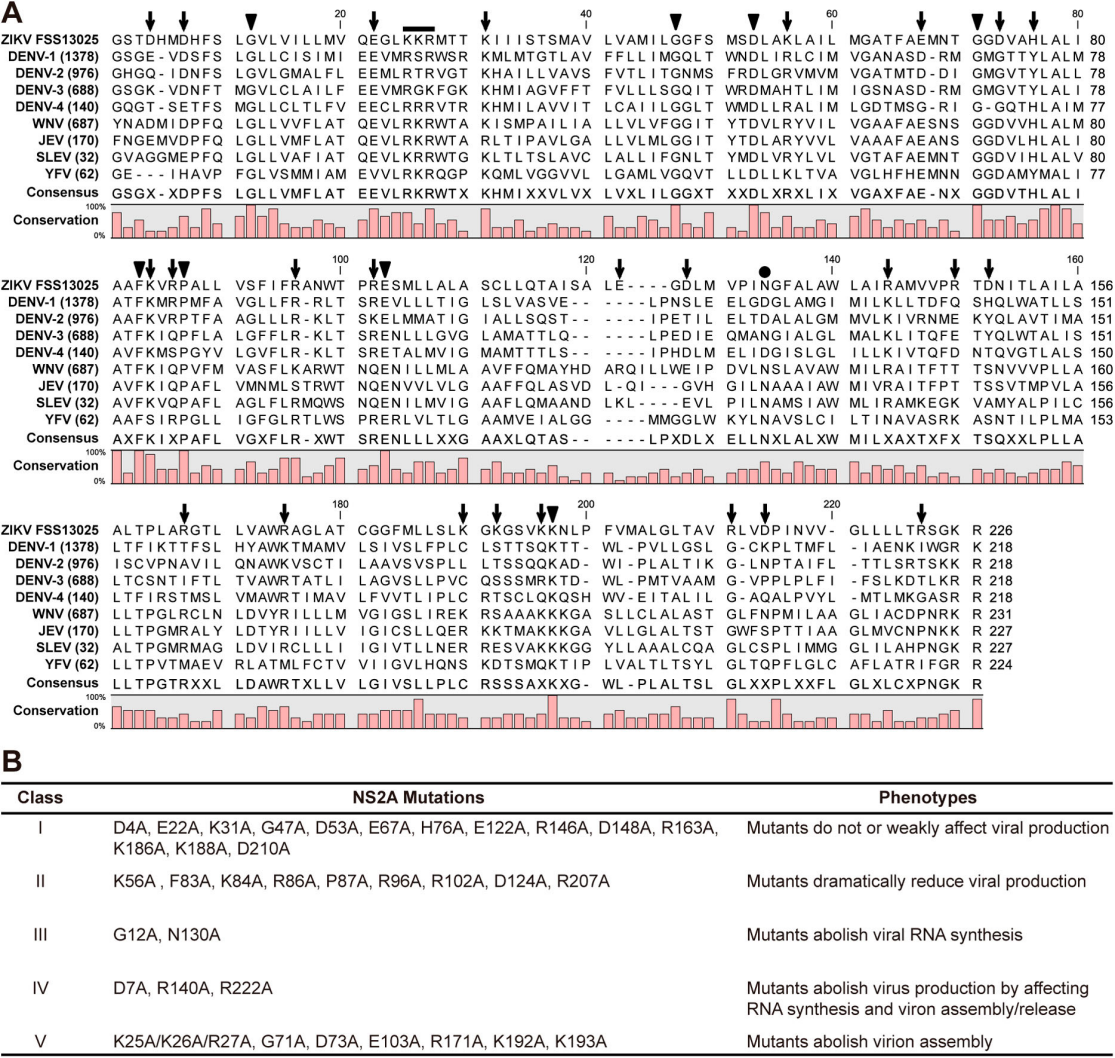
ZIKV NS2A mutagenesis. (A) Sequence alignment of NS2A proteins among ZIKV (strain FSS13025), DENV-1-4 serotypes, WNV, JEV, SLEV, and YFV using CLC Main Workbench software (CLC Bio). Thirty-five amino acids were selected for mutagenesis: (i) All 8 identical non-alanine residues (indicated by triangles), (ii) 25 charged residues (arrows), (iii) a K25/K26/R27 motif (line), (iv) residue N130 (circle). The consensus and conservation percentage are indicated below each amino acids. (B) A summary of ZIKV NS2A mutagenesis results. An infectious cDNA clone and a luciferase replicon of ZIKV strain FSS13025 were used to analyze the function of each amino acid. Based on the replication phenotypes, the 35 mutants could be categorized into five classes as indicated.

To identify critical residues in ZIKV NS2A, we performed a systematic alanine-scanning mutagenesis using an infectious cDNA clone of ZIKV strain FSS13025 [43]. Sequence alignment of NS2A proteins from ZIKV, DENV, WNV, JEV, SLEV, and YFV revealed a high diversity: only 31 out of 218–227 amino acids with a consensus of >80% among different flaviviruses (Figure 1A). A total of 35 residues were selected for mutagenesis: (i) All 8 identical non-alanine residues, including G12, G47, D53, G71, F83, P87, E103, and K193. The amino acid numbering is based on the ZIKV sequence. (ii) All charged residues, including D4, D7, E22, K31, K56, E67, D73, H76, K84, R86, R96, R102, E122, D124, R140, R146, D148, R163, R171, K186, K188, K192, R207, D210, and R222. Charged residues were selected because they likely participate in inter- or intra-molecular interactions; alternatively, they may alter the locations where NS2A would traverse the ER membrane since charged residues are hydrophilic. In addition, NS2A D73H mutation was previously shown to attenuate WNV replication [48]. (iii) A K25/K26/R27 motif that was previously reported to be critical for YFV and DENV virion assembly [35,40]. (iv) Residue N130 that was previously shown to be required for NS1-NS2A cleavage and viral replication in DENV [27,30]. The selected residues were mutated to alanine in the context of ZIKV genomic RNA. Equal amounts of wild-type (WT) and mutant RNAs were electroporated into Vero cells. After electroporation, RNA replication and virus spread were monitored by immunofluorescence assay (IFA); the production of infectious viruses was quantified by plaque assay and RT-PCR. The 35 mutants exhibited distinct phenotypes that could be categorized into five groups (Figure 1B), as detailed in the following sections.

### Class I mutations have no or weak effects on viral replication

**Figure 2.**
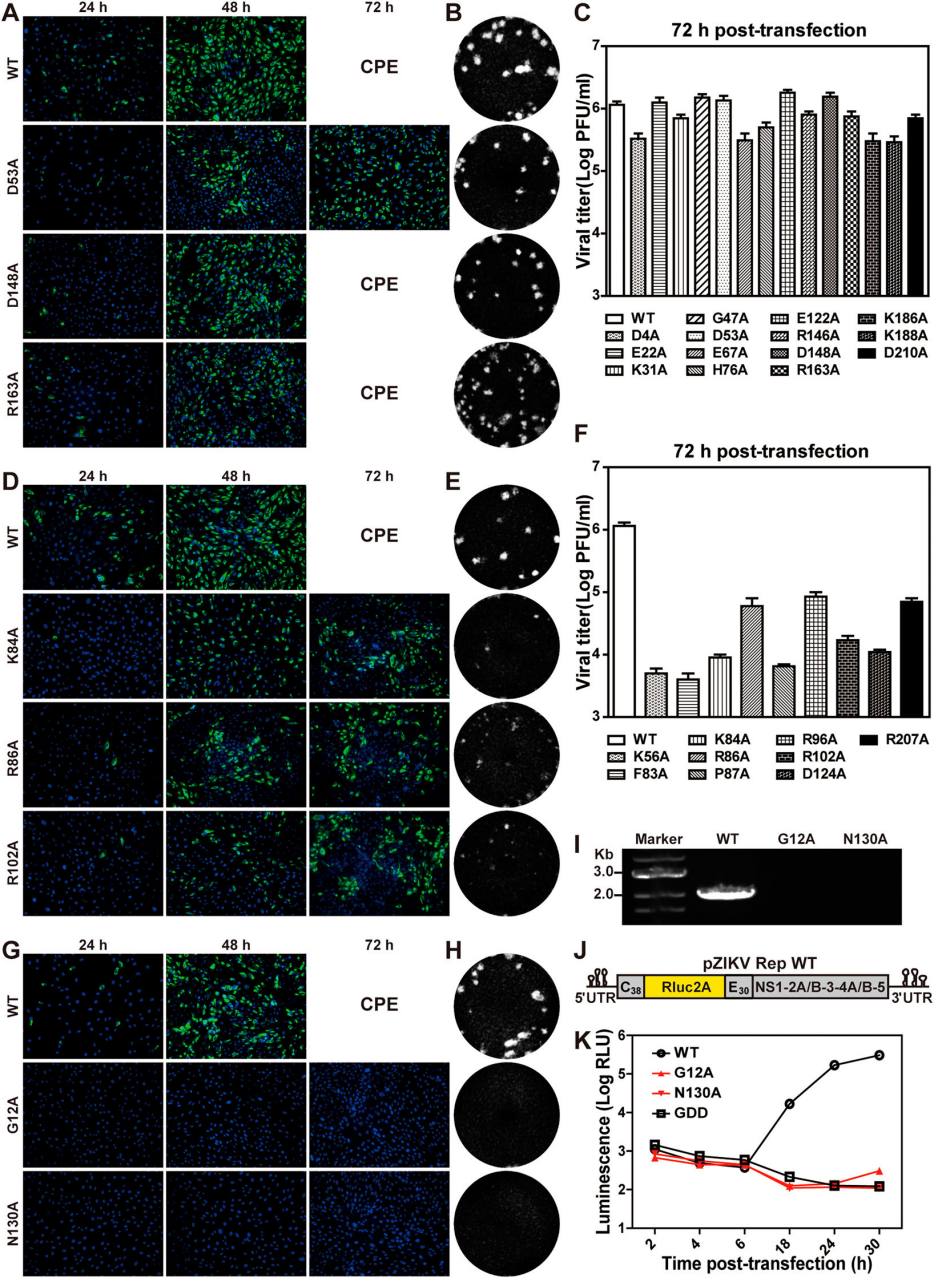
Characterization of class I, II, and III NS2A mutants. The results of representative mutants from class I (A–C), II (D–F), and III (G–K) are shown. (A) Vero cells were electroporated with equal amounts of ZIKV wild-type (WT) or class I mutant genomic RNAs. At indicated time points, the cells were fixed and subjected to IFA. Viral E protein and nuclei were stained with antibody 4G2 (green) and DAPI (blue), respectively. Cytopathic effect (CPE) is indicated at 72 h post-transfection. (B) Plaque morphologies. Recombinant viruses were subjected to plaque assay. Plaques were developed at day 4.5 post-infection. (C) Viral titers of class I mutants at 72 h post-transfection. The mean values from two independent experiments are shown. Error bars indicate the standard deviations. The limit of detection (LOD) of plaque assay is 10 PFU/ml. (D) IFA of class II mutant RNA-transfected Vero cells. (E) Plaque morphology of class II mutant viruses. (F) Viral titers of class II mutant viruses at 72 h post-transfection. (G) IFA of class III mutant RNA-transfected Vero cells. (H) Plaque morphologies of class III mutant viruses. Plaque assay was performed using culture supernatants collected at day 3 (for WT) and day 5 (for G12A and N130A) post-transfection. (I) RT-PCR analysis. At 5 day p.t., the mutant RNA-transfected cells were washed twice with PBS to remove residual input RNAs. At day 8 p.t., the supernatants of G12A and N130A RNA-transfected cells were subjected to RT-PCR. The WT virus (collected at 72 h p.t.) was used as a positive control. (J) A ZIKV luciferase replicon. C_38_ and E_30_ represent the first 38 amino acids of C protein and the last 30 amino acids of E protein, respectively. *Renilla* luciferase: Rluc. Foot-and-mouse-disease virus 2A sequence: 2A. (K) Transient replicon assay. WT, G12A, N130A, and NS5ΔGDD replicon RNAs were electroporated into Vero cells. Luciferase activities were measured at indicated time points. An average of 3 experiments are presented. Bars represent standard deviations.

Fourteen of the 35 NS2A mutant RNAs, including D4A, E22A, K31A, G47A, D53A, E67A, H76A, E122A, R146A, D148A, R163A, K186A, K188A, and D210A, replicated at levels comparable to the WT RNA. These mutants were categorized as class I (Figure 1B). The IFA images and plaque morphologies of three representatives (D53A, D148A, and R163A), as well as the WT, are shown in Figure 2(A and B), respectively. Most class I mutants (e.g. D148A and R163A) yielded comparable E-expressing cells as the WT from 24 to 48 h post-transfection (p.t.). At 72 h p.t., severe CPE occurred among D148A, R163A, and WT RNA-transfected cells. Some class I mutants (e.g. D53A) produced fewer E-positive cells than the WT at 48 h p.t., and spread to infect more cells at 72 h p.t (Figure 2A). All class I mutants produced viruses with plaques similar to the WT virus (Figure 2B). At 72 h p.t., the class I mutants produced infectious titers <4-fold lower than the WT did (Figure 2C). Sequencing of the mutant viruses (collected at 72 h p.t.) confirmed the retaining of the engineered mutations without other changes (data not shown).

### Class II mutations significantly attenuate viral replication

Nine NS2A mutants, including K56A, F83A, K84A, R86A, P87A, R96A, R102A, D124A, and R207A, significantly attenuated viral replication. These mutants were categorized into class II (Figure 1B). Class II RNA-transfected cells produced fewer E-positive cells than the WT did (Figure 2D). At 72 h p.t., only 30–50% of E-positive cells were detected in the class II RNA-transfected cells, whereas the WT RNA-transfected cells exhibited CPE. Class II mutant viruses formed smaller, opaque plaques (Figure 2E), and the transfected cells produced >10-fold lower infectious titers than the WT RNA-transfected cells at 72 h p.t. (Figure 2F). Complete genome sequencing of the class II mutant viruses (collected at 96 h p.t.) did not reveal reversions or any other mutations (data not shown). Because of the weak phenotypes of class I and II mutants, we did not further characterize them.

### Class III mutations are lethal for viral RNA synthesis

Class III includes two mutants, G12A and N130A, which were completely defective in viral RNA synthesis (Figure 1B). The G12A and N130A genomic RNA-transfected cells did not produce any E-positive cells at 24 and 48 h p.t. (Figure 2G). On day 5–8 p.t., no infectious viruses were detected from culture medium by plaque assay (Figure 2H) and RT-PCR (Figure 2I). To confirm the above results, we introduced G12A or N130A mutation into a ZIKV luciferase replicon (Figure 2J). A replication-defective replicon, containing an NS5 polymerase active site ΔGDD mutation, was included as a negative control [44]. Vero cells were electroporated with equal amounts of WT, G12A, N130A, or NS5 ΔGDD replicon RNAs. At 2–6 h p.t., all replicons generated similar levels of luciferase signals, suggesting comparable levels of viral RNA translation and transfection efficiencies (Figure 2K). However, at 18–30 h p.t., G12A and N130A replicons generated low luciferase signals as the non-replicative NS5 ΔGDD replicon did, whereas the WT replicon produced increasing luciferase signals (Figure 2K). These results demonstrate that class III mutations completely abolish viral RNA synthesis.

### Class IV mutations impair both viral RNA synthesis and virion production

**Figure 3.**
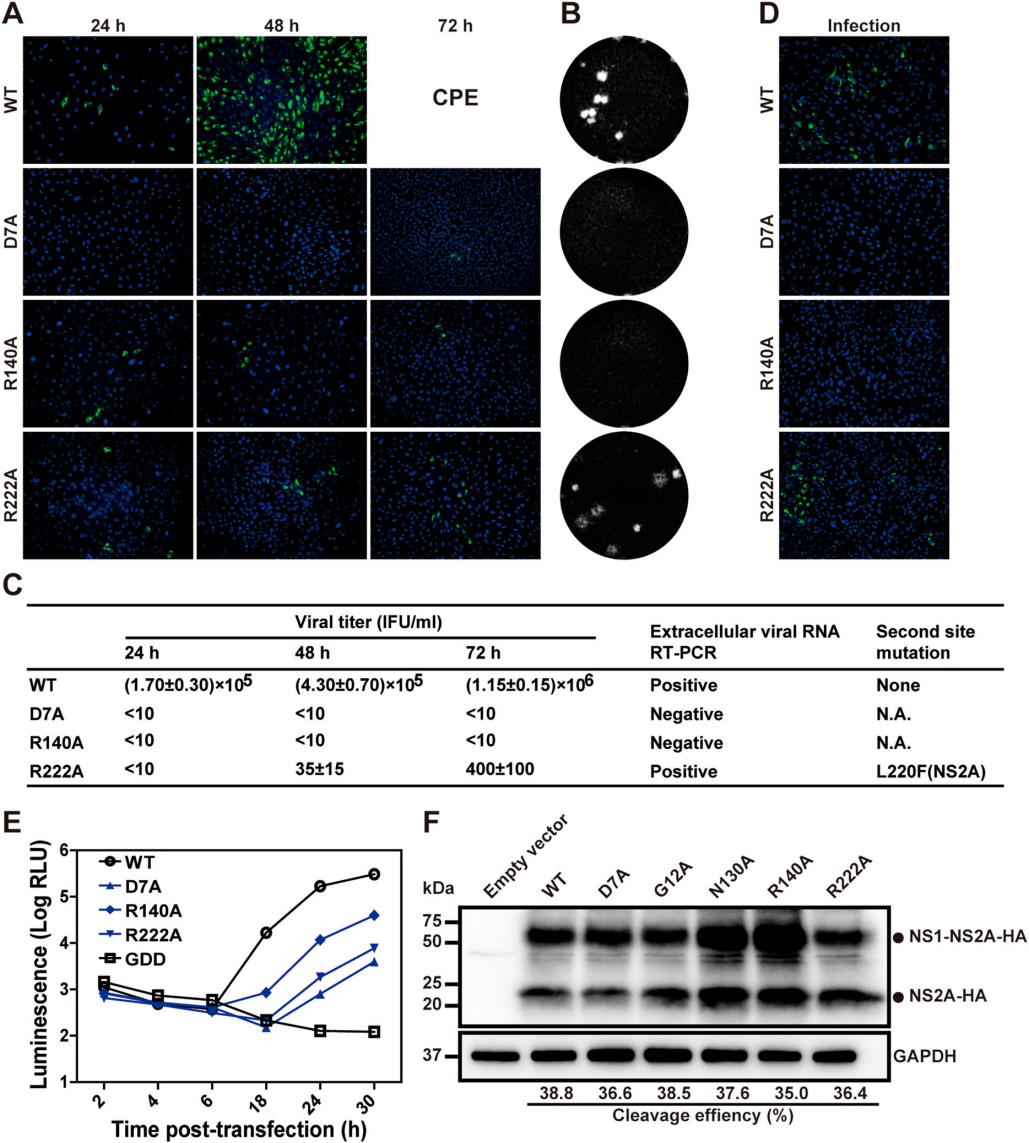
Characterization of class IV NS2A mutants. (A) IFA of transfected Vero cells. (B) Plaque morphologies of WT, D7A, R140A, and R222A viruses. (C) Extracellular viral titers were measured at 24, 48, and 72 h p.t. using plaque assay. At 72 h p.t., extracellular viral RNAs were subjected RT-PCR analysis. The RT-PCR products were sequenced to confirm the engineered mutations and to detect potential second site mutation(s) in the NS2A gene. N.A., not applicable. (D) The supernatants of transfected cells were collected on day 8 p.t. to infect naïve Vero cells. At 24 h p.t., the cells were analyzed for viral E expression by IFA. (E) Transient luciferase replicon analysis. Vero cells were electroporated with ZIKV replicon RNAs and measured for luciferase activities at indicated time points. An average of 3 experiments are presented. Bars represent standard deviations. (F) Analysis of NS2A proteolytic processes. NS2A D7A, G12A, N130A, R140A, and R222A mutations were individually engineered into expression plasmid pXJ-E_24_-NS1-NS2A-HA. After transfecting the plasmids into 293 T cells, total cellular protein samples were harvested at 24 h p.t. and analyzed for NS2A process using Western blot. An HA-tag antibody was used to detect both unprocessed NS1-NS2A-HA and processed NS2A-HA proteins.

Class IV includes three mutants, including D7A, R140A, and R222A (Figure 1B). These mutant genomic RNAs produced fewer E-positive cells in the transfected cells than the WT RNA (Figure 3A). For D7A or R140A mutants, the RNA-transfected cells did not produce infectious viruses, as suggested by plaque assay (Figure 3B) and RT-PCR (Figure 3C). Incubation of naïve Vero cells with culture supernatants from the D7A or R140A transfected cells did not generate any viral E-positive cells (Figure 3D). Replicon analysis showed that D7A or R140A significantly compromised viral RNA synthesis (Figure 3E). Since neither mutants could produce extracellular viruses (Figure 3B and C), and could replicate at attenuated levels inside cells (Figure 3E), D7A and R140A mutations may impair both viral RNA synthesis and virion production.

For R222A, the number of E-positive cells slightly increased in the genomic RNA-transfected cells from 24 to 72 h p.t. (Figure 3A). A low level of infectious virus was detected from the culture medium by plaque assay (Figure 3B) and RT-PCR (Figure 3C). These data were supported by the positive-IFA results after incubating naive Vero cells with the supernatant from the R222A RNA-transfected cells (Figure 3D). Sequencing the virus (collected at 72 h p.t.) revealed that, besides the engineered R222A mutation, the virus had acquired a second site mutation of L220F in NS2A (Figure 3C). Replicon analysis showed that R222A significantly lowered viral RNA synthesis (Figure 3E); no secondary mutation (i.e. L220F) was detected in the replicon RNA at 30 h p.t. (data not shown). These results suggest that (i) R222A mutant is defective in viral RNA synthesis and/or virion assembly, and (ii) a second site mutation L220F in NS2A could rescue the defects.

### Class III and IV mutations do not affect NS2A maturation

Since cleavage between NS1 and NS2A is essential for flavivirus RNA synthesis [30], we examined whether the replication defects of class III and IV mutants were caused by defective maturation of NS2A. To test this hypothesis, we introduced individual mutations into an expression plasmid pXJ-E_24_-NS1-NS2A-HA that encodes a polyprotein containing the C-terminal 24 amino acids of the E protein (serving as a signal peptide to translocate NS1 to ER lumen), NS1, and NS2A with a C-terminal HA tag. After transfecting the plasmids into 293 T cells, the expression of un-cleaved NS1-NS2A-HA intermediate and cleaved NS2A-HA product were analyzed using Western blot (Figure 3F). None of the mutations significantly affected the cleavage efficiencies of NS1-NS2A-HA to NS2A-HA, indicating that the replication defects of class III and IV mutants are not caused by impaired NS2A cleavage.

### Class V mutations block virion assembly

**Figure 4.**
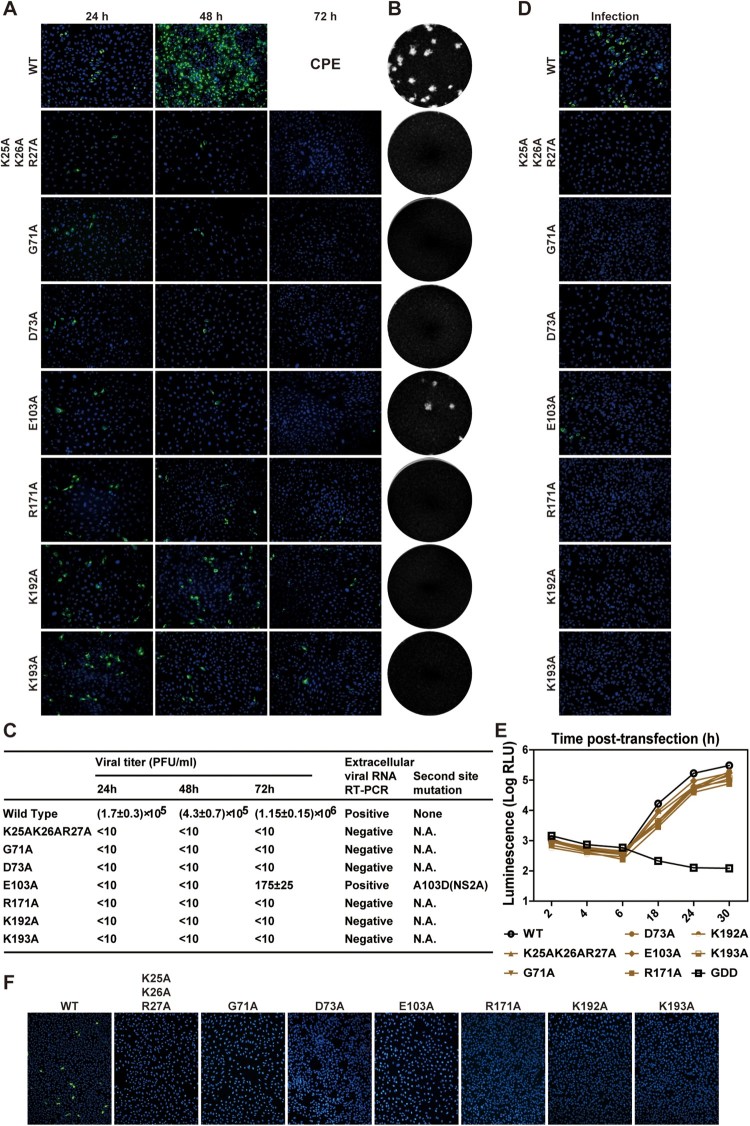
Characterization of class V NS2A mutants. (A) IFA of Vero cells that were transfected with WT or class V genomic RNAs. Viral E-positive cells are indicated in green. (B) Plaque morphologies of class V mutant viruses. Only the WT and E103A mutant generated plaques. (C) Supernatants from the RNA-transfected cells were quantified for viral titers by plaque assay. RT-PCR was also performed on the supernatants at day 3 p.t. for the WT virus and at day 8 p.t. for the mutants. Positive RT-PCR products were sequenced for potential second site mutation(s). N.A., not applicable. (D) Naïve Vero cells were infected with supernatants from the RNA-transfected cells and subjected to IFA for viral E protein expression. (E) Luciferase replicon analysis. Equal amounts of WT and mutant replicon RNAs were electroporated into Vero cells. Luciferase activities were measured at the indicated time points. (F) Analysis of intracellular infectious viruses. Intracellular viruses from ZIKV RNA-transfected cells were harvested at 24 h p.t. as previously described [46]. The harvested samples were used to infect naïve Vero cells. IFA was performed to examine viral E protein expression at 24 h post-infection.

Class V includes G71A, D73A, E103A, R171A, K192A, K193A, and a triple K25A/K26A/R27A. Cells transfected with class V mutant genomic RNAs yielded E-positive cells at 24 h p.t., but the number of E-positive cells did not increase afterward (Figure 4A), suggesting that the mutants are defective in virus spread. No infectious mutant viruses, except E103A, were detected in the supernatants of transfected cells by plaque assay (Figure 4B) or RT-PCR (Figure 4C). No IFA-positive cells were detected after naïve cells were incubated with the supernatants from transfected cells (Figure 4D). Sequencing of the recovered E103A mutant virus revealed an A103D change in the NS2A gene (Figure 4C), suggesting that an E103A-to-A103D substitution is responsible for the rescue of viral replication.

To determine the stage of viral replication that was disrupted by the class V mutations, we analyzed the mutational effect on viral replication in ZIKV replicon (Figure 4E). Compared with the WT replicon, all class V mutants exhibited equivalent levels of viral translation and RNA synthesis (<4-fold difference), suggesting that the mutations only marginally affect viral RNA synthesis. Next, we tested if the class V genomic RNAs could generate intracellular infectious viruses. Incubating naïve Vero cells with WT RNA-transfected cell extracts yielded IFA-positive cells, whereas no IFA-positive cells were detected after incubating naive cells with the mutant RNA-transfected cell extracts (Figure 4F). Altogether, the results suggest that class V mutants are competent in viral RNA replication, but defective in assembly of infectious viruses.

### WT NS2A rescues virion assembly for class V mutants

**Figure 5.**
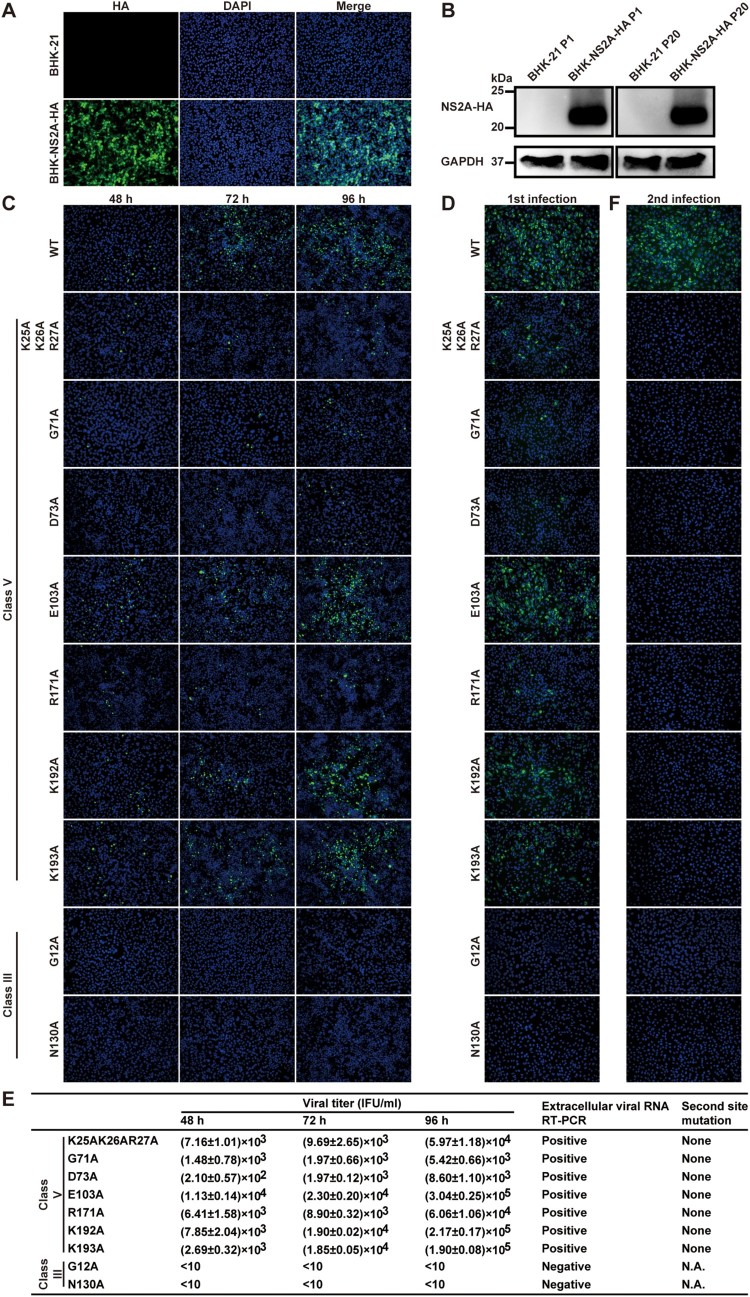
Trans complementation of class III and V mutants. (A) IFA of BHK-NS2A-HA cells. The BHK-NS2A-HA cells constitutively express WT ZIKV NS2A fused with a C-terminal HA-tag. The cells were fixed with paraformaldehyde and stained with an HA-tag antibody. (B) Western blot of BHK-NS2A-HA cells. The expression of WT NS2A-HA from BHK-NS2A-HA cells was detected using an HA-tag antibody. Both passage one (P1) and 20 (P20) BHK-NS2A-HA cells were analyzed. Naïve BHK-21 cells were included as a negative control. (C) WT and class III and V genomic RNAs were electroporated into BHK-NS2A-HA cells. IFA was performed on the transfected cells to detect viral E protein expression. (D) Viruses rescued from the BHK-NS2A-HA cells (harvested at 96 h p.t.) were used to infect naïve Vero cells. The infected Vero cells were subjected to IFA for E protein expression. (E) Extracellular viruses were harvested from the supernatants of RNA-transfected BHK-NS2A-HA cells. The harvested viruses were serially diluted to infect naïve Vero cells in 96-well plates. At 24 h p.i., IFA was performed to quantify the viral titers by counting the number of E-positive cells. The results show averages and standard deviations from triplicate samples. Viral RNAs in the supernatants of transfected BHK-NS2A-HA cells were isolated at 96 h p.t. and subjected to RT-PCR. Positive RT-PCR products were sequenced to confirm the engineered mutations and to identify potential second site change(s). (F) The supernatants of infected Vero cells from (D) were collected at 72 h p.i. to infect naïve Vero cells for a second round. IFA was performed on these cells for viral E-positive cells.

To test *trans* complementation of NS2A mutants, we constructed a BHK-21 cell line that constitutively expressed WT ZIKV NS2A protein with a C-terminal HA-tagged (NS2A-HA). The cell line was constructed by transfecting plasmid pXJ-SPG-C_16_-NS2A-HA encoding a Gaussia luciferase signal peptide (SPG), the C-terminal 16 amino acids of NS1 protein (C_16_), and NS2A-HA, as well as a neomycin resistance gene (*Neo*). The SPG and C_16_ sequences were engineered to enable the correct formation of NS2A membrane topology when expressed in cells. Individual BHK-NS2A-HA cell lines were generated through colony purification under G418 (1 mg/ml) selection. For each BHK-NS2A-HA cell line, all cells were IFA-positive for HA-NS2A expression (Figure 5A). Western blot revealed an expected band of ∼22-kDa band (Figure 5B). The expression level of NS2A-HA did not decrease in the BHK-NS2A-HA cells after 20 passages in culture (4 days per passage; Figure 5B), indicating the stability of the cell line.

We tested *trans* complementation by transfecting class V genomic RNAs to BHK-NS2A-HA cells. The transfected cells developed an increasing number of E-positive cells from 48 to 96 h p.t. (Figure 5C). Culture supernatants were collected at 96 h p.t. to infect naïve Vero cells. The infected cells developed E-positive cells for all class V mutants (Figure 5D), demonstrating the rescue of infectious viruses through *trans* complementation. Quantification of the E-positive cells allowed us to determine the titers of rescued infectious viruses (Figure 5E). Among the class V mutants, E103A, K192A, and K193A exhibited the highest *trans* complementation efficiencies, producing 1.9 × 10^5^–3.04 × 10^5^ IFU/ml viruses at 96 h p.i. (Figure 5E). Sequencing of the rescued virion genomes confirmed the retention of the engineered class V mutations without any other changes in NS2A (Figure 5E).

Next, supernatants from the first round of infection collected at 72 h p.i. (from Figure 5D) were used to infect naïve Vero cells for a second round. In contrast to the first round of infection (Figure 5D), no E-positive cells were detected in the second round of infection for any of the class V mutants (Figure 5F). As a positive control, WT RNA produced high numbers of E-positive cells in both the first and second rounds of infections (Figure 5D and F). Collectively, the results indicate that (i) the defect in virion assembly of class V mutants could be rescued through *trans* supply of WT NS2A protein and (ii) the rescued virions could only infect cells for a single round.

### DENV-2 NS2A could not trans complement ZIKV class V mutants

We tested whether DENV NS2A could rescue class V mutants of ZIKV. Each class V mutant of ZIKV was transfected into a previously established BHK-21 cell line that constitutively expresses WT DENV-2 NS2A protein [30]. For all class V mutants, no increase in E-positive cells or infections viruses was detected from the transfected cells from 24 to 96 h p.t. (data not shown). The results suggest that flavivirus NS2A modulates virion assembly in a virus-type specific manner.

### WT NS2A could not rescue viral RNA synthesis of class III mutants

We tested whether the defect in viral RNA synthesis of class III mutants could be *trans* complemented. To test this possibility, we transfected class III genomic RNAs (G12A and N130A) into ZIKV BHK-NS2A-HA cells. Neither mutants generated any E-positive cells in the transfected BHK-NS2A-HA cells (Figure 5C). Infection of naïve Vero cells with supernatants of the transfected cells did not produce any IFA-positive cells (Figure 5D–F). The results suggest that the RNA synthesis defect of class III mutants could not be rescued through *trans* complementation. Due to the dual defects of class IV mutants in both viral RNA synthesis and virion assembly, we did not pursue *trans* complementation experiments for this class of mutants; such complementation experiments may not reveal clear mechanistic insights as compared with the class III and V mutants.

### Probing the topology of ZIKV NS2A on the ER membrane

**Figure 6.**
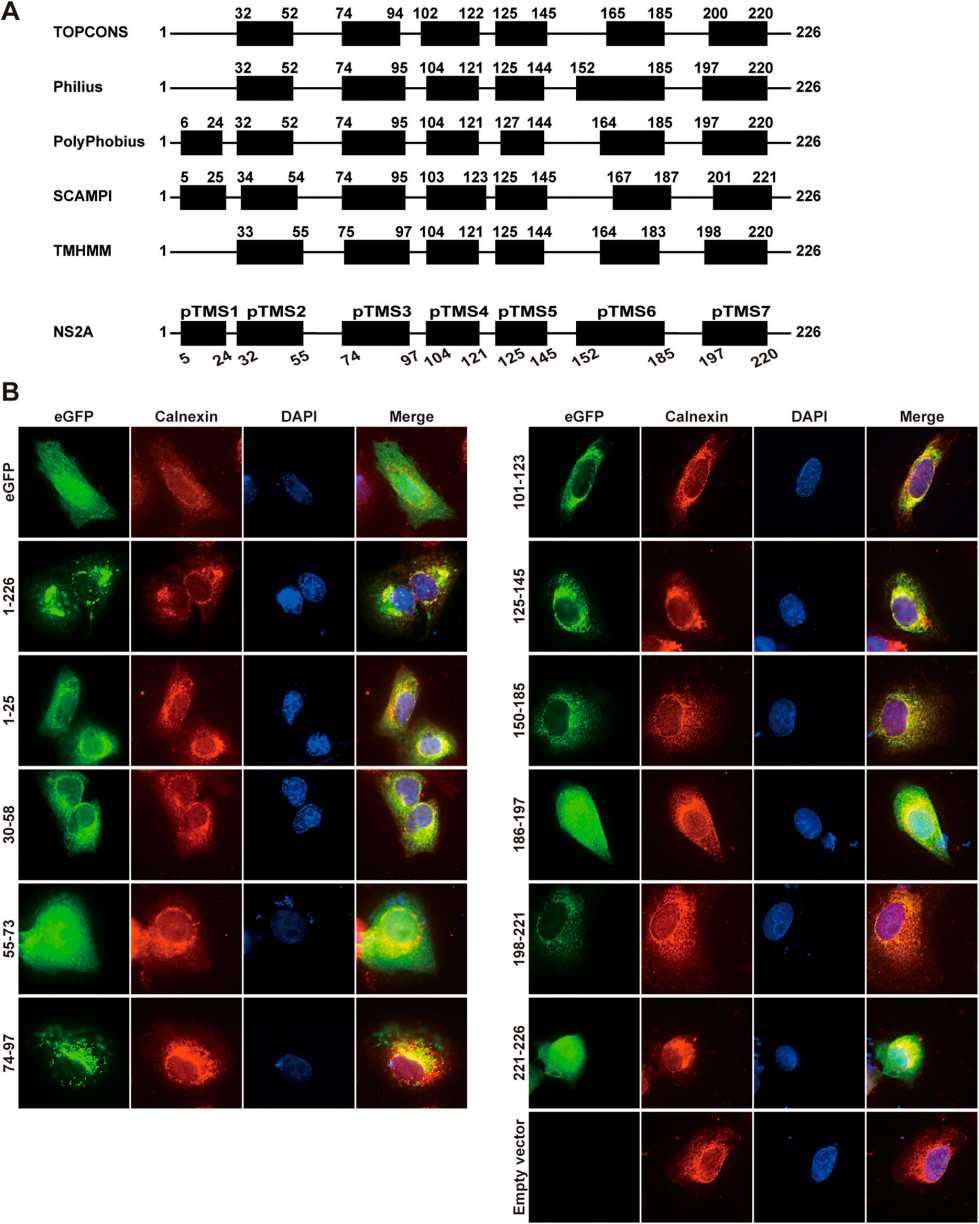
Membrane association of ZIKV NS2A transmembrane segments. (A) Bioinformatics analysis of ZIKV NS2A for transmembrane segments. ZIKV NS2A sequence was analyzed by bioinformatics programme TOPCONS, Philius, PolyPhobius, SCAMPI, and TMHMM. The compiled transmembrane segments are shown at the bottom. Seven predicted transmembrane segments (pTMS) are presented as thick bars. The amino acid positions of ZIKV NS2A are indicated. (B) Membrane association of individual pTMS. Expression plasmid pXJ-eGFP (without NS2A sequence) and pXJ-smNS2A-eGFP (smNS2A represents different NS2A segments) were transfected into Vero cell by using X-tremeGENE 9 DNA transfection reagent (Roche). For each plasmid construct, the NS2A segment is indicated by amino acid positions. At 24 h p.t., IFA was performed to monitor eGFP expression. ER and nuclei were stained with anti-Calnexin antibody and DAPI, respectively. The merged images are shown on the right panels.

The distinct functions of amino acids in viral RNA synthesis and virion assembly prompted us to probe the topology of ZIKV NS2A on ER membrane. Bioinformatics analysis suggests that ZIKV NS2A may form seven transmembrane segments (pTMS1–7; Figure 6A). To examine whether each pTMS associates with the ER membrane, we constructed a panel of plasmids encoding full-length or various NS2A fragments with a C-terminally fused eGFP reporter (Figure 6B). The length of each segment was slightly modified from the bioinformatic prediction to allow some flexibility between pTMS and eGFP. Upon transfection into Vero cells, eGFP alone (without any NS2A fragment) exhibited an even distribution of fluorescence throughout the cell, whereas the full-length NS2A-eGFP (construct 1–226) displayed fluorescence co-localized with calnexin (an ER membrane protein) in the perinuclear region (Figure 6B), indicating that ZIKV NS2A is an integral membrane protein. Expression of seven fragments spanning each pTMS, represented by constructs 1–25 (pTMS1), 30–58 (pTMS2), 74–97 (pTMS3), 101–123 (pTMS4), 125–145 (pTMS5), 150–185 (pTMS6), and 198–221 (pTMS7), exhibited fluorescence patterns that co-localized with calnexin in the perinuclear region (Figure 6B). The results indicate that individual of the seven pTMSes is associated with the ER membrane. In contrast, expression of fragments between different pTMS, represented by constructs 55–73, 186–197, and 221–226, showed fluorescence throughout the cells (Figure 6B), indicating that these fragments are not membrane-associated.

**Figure 7.**
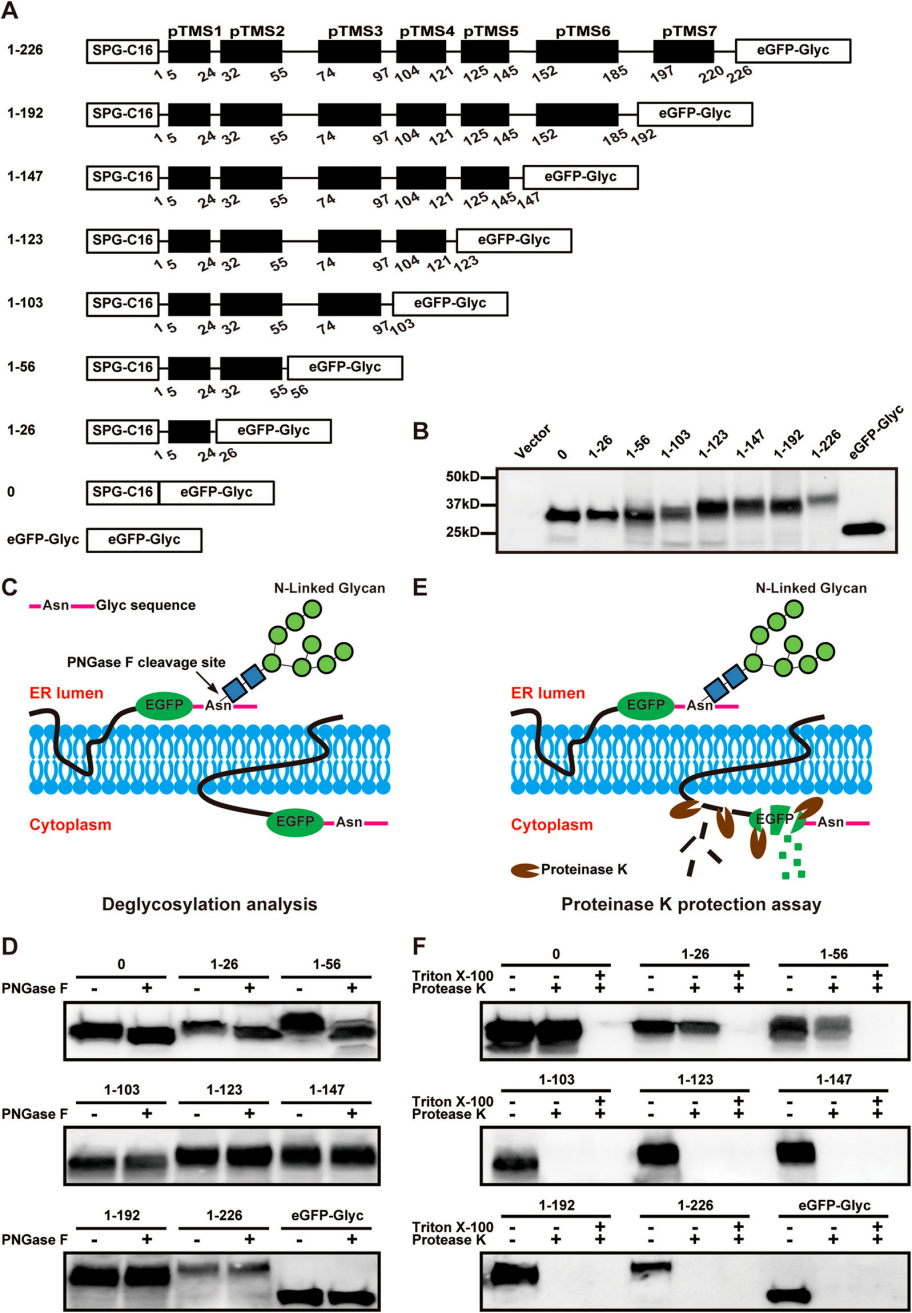
Probing the membrane topology of ZIKV NS2A by in vitro deglycosylation assay and in vitro proteinase K protection assay. (A) Schematic representation of C-terminally truncated ZIKV NS2A proteins. The amino acid positions of each truncated NS2A are indicated. A ‘SPG-C16’ sequence was fused to the N terminus of NS2A to ensure correct translocation of NS2A on the ER membrane. An ‘eGFP-Glyc’ sequence was fused to the C terminus of NS2A to probe its location at cytosol or ER lumen. See text for details. (B) BHK-21 cells were transfected with the expression plasmids described in (A). At 24 h p.t., the expression levels of various NS2A truncates were examined by Western blot using an eGFP antibody. The amino acid positions are indicated for each NS2A truncates. The molecular masses of marker proteins are shown to the left of Western blot image. (C) Diagram of glycosylation of eGFP-Glyc when located at ER lumen (*left*). No glycosylation occurs when eGFP-Glyc is located at cytosol (*right*). The glycosylation residue Asn is indicated. (D) *In vitro* deglycosylation assay. Plasmid-transfected cells were treated with PNGase F or PBS according to the protocol of *in vitro* deglycosylation assay (see Materials and Methods). The samples were examined for glycosylation status by Western blot using an eGFP antibody. (E) Diagram of *in vitro* proteinase K protection assay. When eGFP is located at ER lumen, it is protected from proteinase K cleavage (*left*); when located in the cytosol, eGFP is degraded by proteinase K (*right*). (F) *In vitro* proteinase K protection assay. Cell lysates from plasmid-transfected BHK-21 cells were treated with proteinase K or PBS according to the protocol of *in vitro* proteinase K protection assay (see Materials and Methods). The samples were detected for eGFP by Western blot using an eGFP antibody. Triton X-100 treatment is indicated.

An *in vitro* deglycosylation analysis was performed to determine the orientation of each NS2A pTMS on the ER membrane. A panel of plasmids were prepared to express truncated NS2A proteins that were C-terminally fused with an eGFP followed by an N-glycosylation acceptor peptide (Glyc; Figure 7A). An SPG-C_16_ peptide (Gaussia luciferase signal peptide SPG and the C-terminal 16 amino acids of NS1) was fused to the N terminus of each NS2A-eGFP-Glyc construct to ensure a proper translocation of the expressed protein on the ER membrane. Western blot showed that, upon transfection into BHK-21 cells, all constructs expressed fusion proteins with expected molecular masses (Figure 7B). Because N-glycosylation enzymes reside in the ER lumen, the glycosylation status could be used to indicate the location of the C-terminus of each NS2A-eGFP-Glyc protein (Figure 7C). As a negative control, treatment of EGFP-Glyc (without any viral sequence) with PNGase F (an enzyme that removes high mannose and complex carbohydrates) did not change the mobility of the protein (Figure 7D), indicating a cytosol localization of EGFP-Glyc. As a positive control, PNGaseF treatment increased the mobility of SPG-C_16_-EGFP-Glyc (construct 0), confirming that the C-terminus of this protein resides in the ER lumen. Among the NS2A constructs, PNGase F treatment increased the mobility of fusion proteins 1–26 and 1–56, but not 1–103, 1–123, 1–147, 1–192, and 1–226 (Figure 7D). The results suggest that the N-terminal 56 amino acids of NS2A reside in the ER lumen, and the C-terminal region from residues 103–226 is located in the cytosol.

Next, we performed an *in vitro* proteinase K protection assay [46] to validate the above *in vitro* deglycosylation results. BHK-21 cells were transfected with the same constructs depicted in Figure 7A. At 24 h p.t., membrane fractions from the transfected cells were treated with proteinase K in the presence or absence of 0.5% Triton X-100 and analyzed by Western blot using an eGFP antibody. In the absence of Triton X-100, the eGFP residing in the cytosol would be susceptible to proteinase K digestion, whereas the eGFP residing in the ER lumen would be protected from proteinase K digestion (Figure 7E). As shown in Figure 7F, the eGFPs from constructs 0, 1–26, and 1–56 were protected from protease K digestion, again indicating that the first 56 residues are located in the ER lumen. In contrast, the eGFPs from constructs 1–103, 1–123, 1–147, 1–192, 1–226, and control eGFP-Glyc were completely degraded by the protease K treatment (Figure 7F), supporting that the C-terminal region from residues 103–226 is located in the cytosol. As expected, in the presence of Triton X-100, the eGFPs from all constructs were degraded by proteinase K (Figure 7F). The results of proteinase K assay are in full agreement with those of deglycosylation assay.

### A topology model for ZIKV NS2A

**Figure 8.**
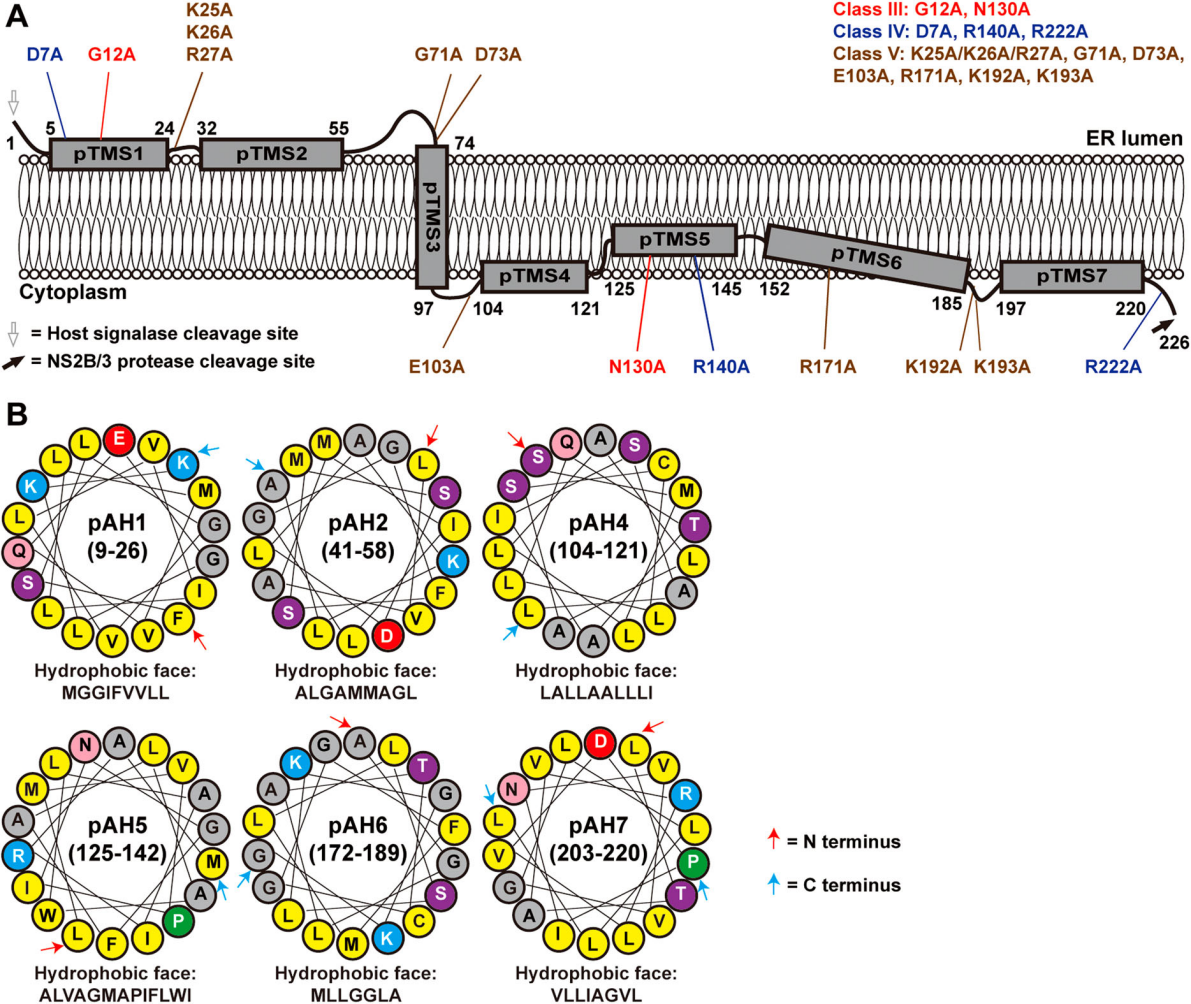
A topological model of ZIKV NS2A. (A) A model of ZIKV NS2A membrane topology. See text for details. Class III, IV, and V mutations are depicted on the topology model. (B) Helical wheel representations of NS2A fragment 9–26, 41–58, 104–121, 125–142, 172–189, and 203–220. The amphiphilic helix regions were analyzed using Heliquest server (heliquest.ipmc.cnrs.fr/). The best 18-residue windows are presented. The amino acids forming the hydrophobic surface of each helix wheel are depicted. Yellow, gray, blue, red, purple, pink, and green circles indicate nonpolar, glycine/alanine, positively charged, negatively charged, polar, amide, and proline amino acids, respectively. The first and last residues in each helical wheel are indicated by a red and blue arrow, respectively.

The above biochemical results support a topology model for ZIKV NS2A on the ER membrane (Figure 8A): (i) The N-terminal region (including pTMS1 and pTMS2) resides in the ER lumen; (ii) the region spanning residues 74–97 (pTMS3) traverses the ER membrane in a lumen-to-cytosol direction; and (iii) the C-terminal region from residues 103–226 (including pTMS4 to pTMS7) is located in the cytosol side. Since pTMS1, pTMS2, pTMS4, PTMS5, PTM6, and pTMS7 associate with ER membraned when expressed individually (Figure 6B), they may interact with the ER membrane in a peripheral manner. In support of this notion, informatics analyses using Jpred 4 and HeliQuest programmes suggest that amino acids 9–26 (pTMS1), 41–58 (pTMS2), 104–121 (pTMS4), 125–142 (pTMS5), 172–189 (pTMS6), and 203–220 (pTM7) may form amphipathic helices with hydrophobic and hydrophilic surfaces (Figure 8B), where the hydrophobic surface may interact with the ER membrane.

## Discussion

Flavivirus NS2A functions in viral RNA replication, virion assembly, evasion of innate immune response, and disease pathogenesis in a virus-specific manner [35]. This is evidenced by its high sequence diversity (Figure 1A), distinct membrane topology [35,46], and association with different disease pathways [41,42]. Thus, it is critical to perform a systematic genetic and biochemical characterization of ZIKV NS2A to delineate its structure and function. Our biochemical results showed that, unlike other flaviviruses, ZIKV NS2A has only a single transmembrane segment that traverses the ER membrane, the other six pTMSes are peripherally associated with the ER membrane (Figure 8A). Our functional analysis revealed five classes of mutants with distinct replication phenotypes, among which class III, IV, and V mutants could not produce infectious viruses through different mechanisms (Figure 1B). The critical mutations from class III to V are not clustered in specific regions of NS2A (Figure 8A), suggesting that multiple domains of NS2A are essential for viral replication.

The membrane topology of ZIKV NS2A is in stark contrast with the topologies of other flavivirus NS2A proteins: DENV-2 NS2A has five membrane-traversing segments [46] and YFV NS2A has three membrane-traversing segments [35]. Interestingly, these topologically diverse flavivirus NS2A proteins are able to converge their functions in viral RNA replication, virion assembly, and evasion of immune response. High resolution, three-dimensional structures of flavivirus NS2A are needed to address this question. It should be noted that the membrane topology of NS2A presented here is in the absence of other viral proteins. In the context of viral replication, the NS2A protein is associated with various viral and cellular proteins that would modulate the structure of NS2A and perform biological functions.

Our mutagenesis study has identified distinct NS2A mutations that selectively abolish ZIKV RNA synthesis (class III) or virion assembly (class V), among which only the virion assembly-defective mutants could be rescued through *trans* complementation (Figure 5). The results suggest that there are two species of NS2A molecules in ZIKV-infected cells. One NS2A species is located at the viral replication complex that could only be formed in *cis*; another NS2A species is located at virion assembly complex that could be formed in *trans* or *cis*. These results are in agreement with the previous studies from DENV and YFV NS2A [30,35,36]. Compared with the viral replication complex, the virion assembly complex is much less well defined for flaviviruses. Previously studies showed that second-site mutations in prM, E, NS2B, and NS3 could rescue the defect of virion assembly caused by DENV-2 and YFV NS2A mutations, suggesting that these viral proteins participate in virion assembly. Besides the genetic evidence, the role of NS3 in virion assembly is also supported by (i) mutations in the NS3 helicase domain alone affect YFV assembly [49], (ii) the helicase domain of DENV or YFV NS3 directly binds to NS2A [35,36], (iii) the cleavage between C and prM is tightly modulated by NS2B/NS3 protease and cellular signalase during YFV and Murray Valey encephalitis virus assembly [50–52]. These data, together with the result that recombinant NS2A of Kunjin binds to viral 3’UTR RNA [29], suggest a flavivirus assembly model. During flavivirus assembly, the NS2A protein recruits NS2B/NS3 through binding to the NS3 helicase domain. Once the NS2A-NS2B/NS3 complex reaches the virion assembly site, the NS3B/NS3 protease cleaves the C protein from the C/prM/E polyprotein. The C protein assembles onto genomic RNA (presented by NS2A) to form nucleocapsid, which is subsequently enveloped by prM/E complex to form virions. Since flavivirus assembly has to be coupled to viral RNA replication [53], the newly synthesized genomic RNA has to be transported from the replication site to the virion assembly site [54], possibly through the NS2A protein.

The *trans* complemented class V mutants could potentially be used as vaccine candidates, as recently reported for DENV-2 [36]. Since the *trans* complemented virions are able to infect cells for only a single cycle, such vaccine platform is safer than the multi-round infectious live-attenuated vaccines [55,56]. This is particularly important for ZIKV vaccine when immunizing immune-compromised individuals and pregnant women [57]. However, for development of class V mutants as a vaccine platform, the *trans* complementation efficiency needs to be improved to higher titers before they could be tested for *in vivo* efficacy.

In summary, we report the genetic and biochemical characterization of ZIKV NS2A protein. The results indicate that different flavivirus NS2A proteins have distinct membrane topologies, but converge to function in viral RNA synthesis and virion assembly. Together with previous results, we propose a model for flavivirus assembly in which NS2A plays a central role in mediating viral structural and nonstructural proteins as well as genomic RNA during virion formation.
